# Translesion DNA Synthesis Across Lesions Induced by Oxidative Products of Pyrimidines: An Insight into the Mechanism by Microscale Thermophoresis

**DOI:** 10.3390/ijms20205012

**Published:** 2019-10-10

**Authors:** Ondrej Hrabina, Viktor Brabec, Olga Novakova

**Affiliations:** 1 Czech Academy of Sciences, Institute of Biophysics, Kralovopolska 135, CZ-61265 Brno, Czech Republic; 2 Department of Biophysics, Faculty of Science, Palacky University, Slechtitelu 27, CZ7837177146 Olomouc, Czech Republic

**Keywords:** oxidized nucleotides, 2’-deoxyribo-5-hydroxyuridin, 2’-deoxyribo-5-hydroxymethyl- uridin, translesion DNA synthesis, DNA polymerases, microscale thermophoresis

## Abstract

Oxidative stress in cells can lead to the accumulation of reactive oxygen species and oxidation of DNA precursors. Oxidized nucleotides such as 2’-deoxyribo-5-hydroxyuridin (HdU) and 2’-deoxyribo-5-hydroxymethyluridin (HMdU) can be inserted into DNA during replication and repair. HdU and HMdU have attracted particular interest because they have different effects on damaged-DNA processing enzymes that control the downstream effects of the lesions. Herein, we studied the chemically simulated translesion DNA synthesis (TLS) across the lesions formed by HdU or HMdU using microscale thermophoresis (MST). The thermodynamic changes associated with replication across HdU or HMdU show that the HdU paired with the mismatched deoxyribonucleoside triphosphates disturbs DNA duplexes considerably less than thymidine (dT) or HMdU. Moreover, we also demonstrate that TLS by DNA polymerases across the lesion derived from HdU was markedly less extensive and potentially more mutagenic than that across the lesion formed by HMdU. Thus, DNA polymerization by DNA polymerase η (polη), the exonuclease-deficient Klenow fragment of DNA polymerase I (KF^–^), and reverse transcriptase from human immunodeficiency virus type 1 (HIV-1 RT) across these pyrimidine lesions correlated with the different stabilization effects of the HdU and HMdU in DNA duplexes revealed by MST. The equilibrium thermodynamic data obtained by MST can explain the influence of the thermodynamic alterations on the ability of DNA polymerases to bypass lesions induced by oxidative products of pyrimidines. The results also highlighted the usefulness of MST in evaluating the impact of oxidative products of pyrimidines on the processing of these lesions by damaged DNA processing enzymes.

## 1. Introduction

**Figure 1:**
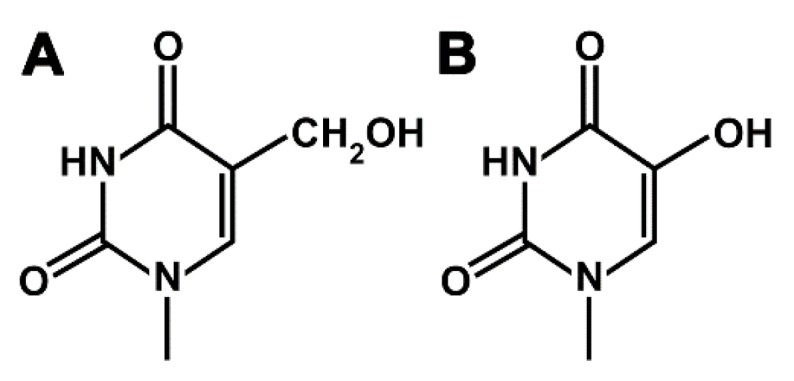
Structures of 5-hydroxymethyluracil (HMdU) (**A**) and 5-hydroxyuracil (HdU) (**B**).

Oxidized nucleosides represent one of the main classes of damage induced in DNA by physical and chemical agents [1]. Examples of such oxidized nucleosides are 2’-deoxyribo-5-hydroxyuridin (HdU) and 2’-deoxyribo-5-hydroxymethyluridin (HMdU) (Figure 1). Both HdU and HMdU represent the most common oxidized pyrimidine 2’-deoxyribonucleosides found in DNA generated by reactive oxygen species (ROS), constantly formed by endogenous processes such as aerobic respiration and phagocytosis and by exposure to ionizing radiation [2,3,4]. The difference between the structures of HdU and HMdU is only seen in the absence of the methylene (CH_2_) group in HdU. Despite this relatively small difference in structure, the presence of HdU and HMdU in double-helical DNA is detrimental to the cell in different ways. HdU is produced by the oxidative de-amination of cytosines by reactive oxygen species [2,3]. HMdU is formed via oxidation of thymine and is also produced through enzymatic oxidation of thymine [5]. Therefore, incorporation of dAMP opposite HdU in template DNA should be regarded as a mutagenic or premutagenic event because the base-pairing of A (primer DNA):HdU (template DNA) will lead to a base pair of A:T in the next round of DNA replication. These events will result in a G:C to A:T transition mutation. This specificity of mutation (C to T by HdU) is consistent with the results published previously [6]. In contrast, HMdU is generated by methylation of thymine in DNA by the ten eleven translocation (TET) enzyme. Incorporation of dAMP opposite HMdU in template DNA should be regarded as a “non-mutagenic” event because A:HMdU will lead to A:T (the same base pair as the original one) in the next round of DNA replication.

One of the most notable biochemical differences between HdU and HMdU found in DNA includes the incapability of the former to distort the molecule of double-helical DNA. As a consequence, replicative DNA polymerases readily bypass the HdU lesion, preferentially incorporating dAMP [2,7]. HdU found in double-helical DNA is potentially mutagenic [6,7,8] and not cytotoxic [9]. Interestingly, several enzymes from human cells capable of removing HdU lesions from DNA have been described [3]. This result suggests that the HdU lesion has significant mutagenic potential. Additionally, HdU can form stable base pairs with dA, dG, dC, and thymidine (dT) residues in a DNA duplex, providing a basis for the in vivo incorporation of HdU during DNA replication [3].

The presence of HMdU in double-helical DNA does not distort its conformation as well [10], although it enhances double-helical DNA flexibility [11] and perturbs DNA–protein interactions [12,13]. Moreover, it is not a block for DNA or RNA polymerases [14] but does not miscode [15]. Additionally, it has also been proposed that HMdU present in double-helical DNA might cause genomic instability as it can form potentially mutagenic lesions and destabilize double-helical DNA [16]. However, this formation of mutagenic and DNA destabilizing lesions is not due to the intrinsic properties of HMdU, but is as a consequence of its removal by DNA repair enzymes [17,18] and subsequent errors arising during its repair [19]. Notably, HMdU is one of several recently discovered epigenetic modifications [11,13]. This pyrimidine derivative is, in contrast to HdU, cytotoxic [20] and has antiviral activity [21].

Hence, one explanation of the different effects of HdU and HMdU on cells might also be connected with the different mechanisms of replication fork bypass of HdU and HMdU residues in double-helical DNA. Mechanisms for replication fork bypass of sites in DNA damaged by physical and chemical agents have been extensively investigated, but they are not entirely understood. For instance, the mechanisms of translesion DNA synthesis (TLS) across various DNA lesions by several DNA and RNA polymerases have been studied extensively (see, e.g., [22,23,24,25,26,27,28,29,30]), but the energetic aspects underlying the replication fidelity and the long-range effects of the lesion on translesion synthesis across HdU or HMdU have not been entirely resolved. This information may be helpful since, for instance, the enthalpy involved in an enzyme-directed insertion event has been shown similar to that required in base pair addition, as predicted by nearest neighbor data derived from melting studies [31]. Ultimately, energy data repositories of sufficient density and diversity may yield predictive capacities for assessing the thermodynamic origins of different cellular “downstream” processes that result from DNA modification due to the presence of HdU and HMdU. The comparative studies of the DNA lesions of HdU and HMdU may begin to explain the role of functional groups in the DNA base residues in creating specific local alterations of flexibility and/or hydrophobicity and subsequent downstream cellular processes leading to DNA and cell damage.

The goal of this study, performed in cell-free media, has been to show whether there are correlations between the biophysical properties (conformational and thermodynamic characteristics) of HdU and HMdU DNA lesions on the one hand and an ability of these lesions to block DNA polymerization and/or cause a mutation on the other hand.

## 2. Results and Discussion

### 2.1. Determination of Thermodynamic Parameters of Duplex DNA Constructs with Nucleotide Misincorporation Opposite HdU and HMdU Lesions by Microscale Thermophoresis

The mutagenic effects of HdU and HMdU and some details of the mechanisms of these effects have been determined by molecular biology and biochemical methods [2,7,13,32]. However, the energetics involved in these mechanisms, such as the thermodynamic aspects of the replication fidelity of the oxidative products of pyrimidines, have not been examined. Therefore, we characterized energetics associated with the mutagenic effects of HdU and HMdU in the quest to contribute to understanding (1) the nature of the forces that dictate and control TLS across HdU and HMdU lesions; and (2) why the damage induced by the HdU and HMdU lesions affects polymerase fidelity and processivity differently. With this aim in mind, we used microscale thermophoresis (MST). MST is based on the directed motion of molecules in microscopic temperature gradients and enables the quantitative analysis of molecular interactions in solution at the microliter scale. It should be pointed out that all previous studies (aimed at exploration of the thermodynamic aspects of the TLS across DNA lesions) were performed using experimental approach of a simulated TLS employing differential scanning calorimetry (DSC). DSC was introduced by Liang and Cho [33] and has been used by us and others in several previously published studies [34,35,36,37,38,39,40,41]. Thus, the present study represents the first attempt to use MST in such studies.

**Figure 2:**
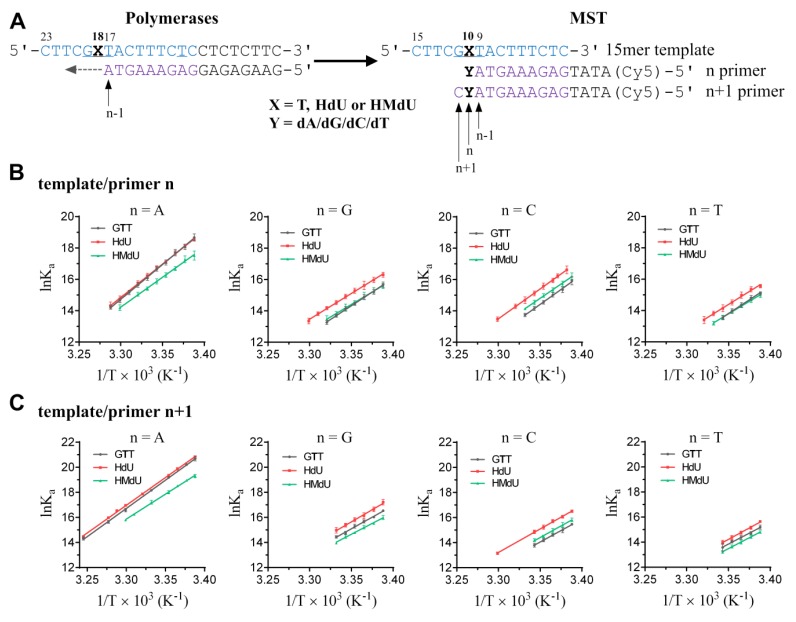
Microscale thermophoresis (MST) determination of the thermodynamic parameters of DNA constructs with nucleotide misincorporation opposite thymine and its derivatives. (**A**) Sets of sequences of successive template-primers designed to simulate translesion DNA synthesis (TLS), where X indicates thymine or its HdU or HMdU variant, and Y is the site of a mismatch. n−1: position one nucleotide before lesions, n: position opposite the lesions, n+1: position one nucleotide behind lesions. The identical 15mer template sequence is in blue and complementary primers in violet color. (**B**,**C**). van’t Hoff plots of the n or n+1 primer-template hybridization reactions. *K*_d_ values were calculated for each temperature by fitting the T-Jump or thermophoresis signal and plotted as ln(*K*_a_ = 1/*K*_d_) vs. 1/*T* (K). ∆*H* was obtained from the slope m of the linear fit as m = −*H^o^/R*. Under the assumption that ∆*H* is constant in the relatively small linear range of the van’t Hoff plot, ∆*S* was directly derived from the plot as y(0) = ∆*S*^o^*/R*. The universal gas constant *R* = 8.314 J K^−1^mol^−1^. Data are means (±SD) from at least two different experiments; coefficient of determination *r^2^* ≥0.99.

We used MST for the thermodynamic analysis of various types of DNA duplexes. We hybridized 15-bp DNA templates, unmodified or containing the HdU or HMdU lesion in the same sequence context: 5‘--- G**T**T ---3′ or 5‘--- G**X**T ---3′ (X = HdU or HMdU) with 10mer or 11mer matched or mismatched primers, n and n+1, prolonged on the 5′ site by four nucleotides to eliminate any effects of Cy5 fluorophore on the hybridized DNA duplex (the nucleotide sequences are shown in Figure 2A). Thus, these duplexes were used to simulate to TLS across and one nucleotide past HdU or HMdU lesions. In quantifying the energetics of the interactions of the DNA templates and primers, a convenient and intuitive measure is the equilibrium dissociation constant *K*_d_. The dissociation constant *K*_d_ was calculated from the differing MST signals for single-stranded and double-stranded DNA as described in the previously published study using the software provided by the manufacturer for the evaluation of MST experiments [42].

Inspection of the thermodynamic data (Table 1, Table 2, Table 3 and Table 4) reveals that the *K*_d_ values of the dissociation reactions at the ambient temperature were in the low nM to pM range, but shifted towards significantly higher values with increasing temperature. Moreover, the experiments were performed with two primer sets (n and n+1), which differed in length, carrying a single mismatch (Figure 2A). Unsurprisingly, the hybridization affinity of the templates and primers was reduced if the primers contained the mismatched nucleotides (Table 1, Table 2, Table 3 and Table 4). Additionally, the hybridization affinity and thermodynamic stability of all mismatched duplexes containing HdU lesions in the template strand increased in comparison with the duplexes containing the control G**T**T template strand or the template containing HMdU lesions. 

After determining the *K*_d_ values of DNA hybridization for each temperature, van’t Hoff plots (ln(*K*_a_) vs. 1/*T* (Figure 2B,C), where *K*_a_ is the association constant which equals 1/*K*_d_), were used to deduce the thermodynamic parameters ∆*H* and ∆*S* (Table 1, Table 2, Table 3 and Table 4) as described in the Experimental section and in the previously published work [42].

The mismatched template/n or n+1 duplexes were enthalpically destabilized in comparison with the matched duplexes in the case of all three DNA template sets (the unmodified template or template containing the HdU or HMdU lesion (Figure 2B,C)). Moreover, while replacement of dT in the G**T**T control template by HdU stabilized the mismatch-duplexes, the replacement by HMdU stabilized these duplexes only very slightly (in the latter case, the Gibbs free energies (Δ*G*_0_^310^) were similar to those obtained for the duplexes formed from the unmodified control G**T**T templates).

### 2.2. DNA Synthesis by DNA Polymerases across Pyrimidine Oxidative Products HdU and HMdU

We also investigated in the present work DNA polymerization in a cell-free medium by three DNA polymerases: DNA polymerase η (polη, a xeroderma pigmentosum variant involved in the DNA repair by translesion synthesis), the endonuclease-deficient Klenow fragment of DNA polymerase I (KF^–^), and reverse transcriptase from human immunodeficiency virus type 1 (HIV-1 RT). We used the templates site-specifically modified by unique HdU or HMdU. The results of these experiments were compared with DNA polymerization using the control template containing canonical thymidine (dT) instead of HdU or HMdU. Hence, we went on to investigate the effect of replacement of hydrophobic 5-methyl group in the pyrimidine ring by less hydrophobic 5-hydroxymethyl or hydrophilic 5-hydroxyl groups on the process of DNA polymerization across these pyrimidine residues and to correlate these data with thermodynamic characteristics of the corresponding DNA duplexes.

The DNA polymerization was examined by polη, KF^–^, and HIV-1 RT because they can provide a basis for making similar comparisons with other prokaryotic or eukaryotic polymerases. In eukaryotes, the Y-family DNA polymerases (η, ι, κ, and Rev1) replicate across DNA lesions [43]. The extensive genetic, biochemical, and structural studies that have been carried out on Klenow fragment make it an ideal model system for investigating the molecular mechanism of template-directed DNA synthesis and the way in which the polymerases interact with DNA [44]. Thus, the DNA polymerase I class of enzymes has served as the prototype for studies on structural and biochemical mechanisms of DNA replication [45,46]. The exonuclease-deficient Klenow fragment (KF^–^) was selected here because translesion synthesis-proficient DNA polymerases of the X or Y families share some common properties, including lack of associated 3′ to 5′ exonuclease proofreading activity, and the proofreading mechanism itself may introduce effects more dependent on the lesion type [47]. We also used HIV-1 RT in these studies, which possesses DNA template-dependent DNA polymerase activity, but relatively low processivity and fidelity [48].

**Figure 3:**
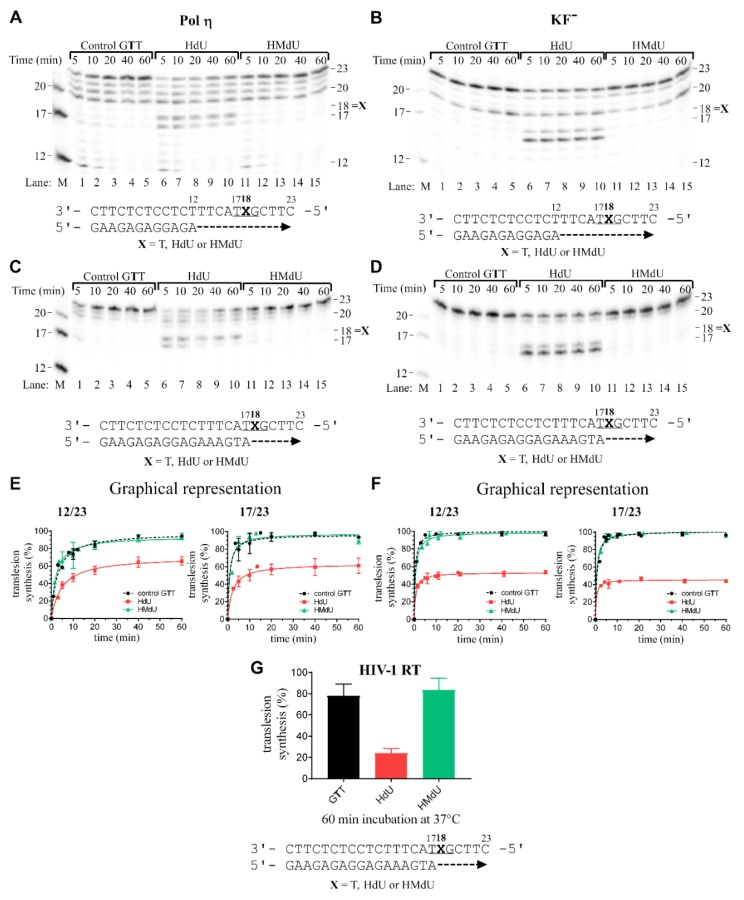
Translesion DNA synthesis by human DNA polymerase η (polη), the exonuclease-deficient Klenow fragment of DNA polymerase I (KF^–^), and reverse transcriptase from human immunodeficiency virus type 1 (HIV-1 RT) on templates containing a site-specific thymine lesion. Primer extension activity of polη (A, C, E), KF^–^ (B, D, F), and HIV-1 RT (G). (**A**,**B**,**C**,**D**) Representative images of the products of DNA polymerases reactions resolved on 15% polyacrylamide (PAA) gels. 12mer (gap-primer for “running start” experiments) or 17mer (no gap-primer for “standing start” experiments) DNA primers were complementary to the 3’ termini of the 23mer templates. The experiments were conducted using the 12-mer/23-mer (panels **A**,**B**) or 17-mer/23-mer primer/template duplexes (panels **C**,**D**) for the various times (time points of 5–60 min are shown above the gels) using undamaged template (panels **A**,**B**,**C**,**D**, lanes 1–5), the template containing HdU or HMdU instead of thymine at the 5′-G**X**T sequence (panels **A**,**B**,**C**,**D**, lanes 6–10 or lanes 11–15, respectively). The pause sites and position of thymine modification (the product lengths) are shown on the right side of the gels. Lane M: DNA markers. The nucleotide sequences of the templates and the primers are shown at the bottom of panels **A**,**B**,**C**,**D**, and **G**. (**E**,**F**) Densitometric evaluations of the amount of synthesis past undamaged or modified templates. Left panels: “Running-start” synthesis. Right panels: “Standing-start” synthesis. The graphs show the time dependence of the inhibition of DNA synthesis on undamaged (control) template (full circles), DNA containing HdU (full squares), and DNA containing HMdU (full triangles). Translesion DNA synthesis by HIV-1 RT is plotted in graph G for the “steady-state” after 60 min incubation. Data are means (±SEM) from three different experiments. For some points, the error bars are smaller than the size of the symbol.

In order to assess the translesion replication capacity of polη, KF^–^, and HIV-1 RT, we first investigated their ability to elongate a 5′-^32^P-labeled 12mer or 17mer primer annealed to 23mer templates containing control canonical dT (Figure 3A–D, lanes 1–5) or to 23mer templates containing HdU or HMdU pyrimidine derivatives, respectively (depicted in Figure 3A–D, lanes 6–10 or 11–15, respectively; the corresponding nucleotides in these templates are marked by bold letters also throughout the text). These experiments were carried out in the presence of all four 2’-deoxyribonucleotide-5‘-triphosphates (dNTPs). The 3’ thymine/lesion (HdU or HMdU) involved in the G**T**T sequence on the template strand was located at its 18th position from the 3’ terminus (positioning the 3’-end of the primer five bases before the modified base in the template strand in “running start” experiment or just before lesion in “standing start” experiment) (Figure 3A–D,G). The newly synthesized DNA products were resolved by denaturing polyacrylamide (PAA) gel electrophoresis and visualized by radiography.

DNA polymerization through the single HdU or HMdU lesion of pyrimidines on the template by polη or KF^–^ in the presence of all four dNTPs was stopped at various time intervals. In the case of HIV-1 RT, only 60 min incubation in “standing start” experiment was performed (12mer primer used in “running start” experiment seems too short). Polymerizations from both 12mer and 17mer primers proceeded rapidly up to the nucleotide corresponding to the sites one before and opposite to HdU lesion, such that the 17mer and 18mer products, respectively, accumulated to a significant extent (Figure 3A–D, lanes 6–10 and 3E–G). Contrastingly, the polymerization across HMdU lesion by all three DNA polymerases proceeded with a similar rate as was that across the control canonical dT (Figure 3A–D, lines 11–15 and 3E–G). These results confirm that HMdU incorporated in DNA does not represent a significant block to the investigated DNA polymerases whereas HdU incorporated in DNA is markedly less tolerated by DNA polymerases tested in the present work.

**Figure 4:**
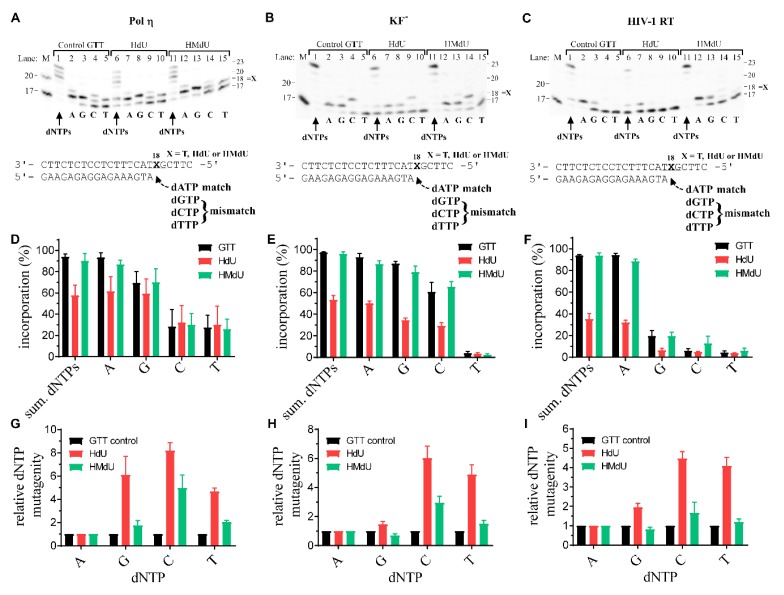
The replication fidelity of polη, KF^–^, and HIV-1 RT. The capacity to elongate a 5′-^32^P-labeled 17mer primer annealed to the unmodified 23mer templates (panels **A**, **B**, **C**, lanes 1–5) or to the 23mer templates containing HdU or HMdU thymine derivatives, respectively, as depicted in panels **A**, **B**, **C** in lanes 6–10 or 11–15, respectively (the corresponding nucleotides in these templates are marked by bold letters at the bottom of gels) in the presence of all four deoxyribonucleotide 5‘-triphosphates (dNTPs) or complementary dATP or non-complementary nucleotides individually. Lane M: DNA markers. The newly synthesized DNA products were resolved by denaturing 15% PAA gel electrophoresis and visualized by radiography. The amount of synthesis was defined as the amount of radioactivity corresponding to the products of incorporation, (18–23) nucleotides long, on the 5’ side of the template strand and beyond divided by the total radioactivity in the respective lane (panels **D**, **E**, **F**) for each thymine derivative. Relative dNTP mutagenicity (panels **G**, **H**, **I**) was calculated as a ratio of the respective misincorporation frequency *f* = (*K*_cat_*/K*_m_)_incorrect_/(*K*_cat_*/K*_m_)_correct_ for the oligonucleotides with thymine derivatives and control oligonucleotide with thymine. Data are means (±SD) from three different experiments.

In order to assess the replication fidelity of polη, KF^–^, and HIV-1 RT, we investigated their capacity to elongate a 5′-^32^P-labeled 17mer primer annealed to the 23mer templates containing a single, control canonical dT (Figure 4A–C, lanes 1–5), HdU or HMdU (depicted in Figure 4A–C, lanes 6–10 or 11–15, respectively). These experiments were performed in the presence of all four dNTPs or only complementary dATP or one of the other three, non-complementary dNTPs. The newly synthesized DNA products were resolved by denaturing PAA gel electrophoresis and visualized by radiography. Synthesis (%) after 60 min incubation is shown (Figure 4D–F). To determine the fidelity of DNA polymerases, we conducted single-nucleotide incorporation assays—steady-state kinetic analysis (Tables S1–S3 and Figures S3–S5). Not surprisingly, incorporation of the matched dAMP was preferred by all three investigated polymerases; the presence of HdU caused the highest nucleotide misincorporation (Figure 4, Tables S1–S3). The HMdU derivative also increased a misincorporation compared to the control, unmodified G**T**T template, but much less than the presence of HdU (Figure 4, Tables S1–S3). In aggregate, the data in Tables S1–S3 show that the correct dAMP is incorporated opposite dT or pyrimidine oxidative products with efficiency which is considerably higher than that of the other incorrect nucleotides. Nevertheless, our data do not exclude eventuality that the polymerases elongate the primer incorporating dAMP opposite the HdU and HMdU lesions less efficiently than opposite to dT.

In contrast to HIV-1 RT, under the same experimental conditions, DNA polymerizations by polη or KF^–^ using the control template or template containing HMdU formed a relatively high extent of mismatches with dGTP. It may be suggested that the latter observation is a consequence of structural similarities of dATP and dGTP. With regard to the misincorporation of dCMP or dTMP, the presence of HMdU lesions caused a significant accrual of the mismatches (Figure 4). The efficiency (*K*_cat_/*K*_m_) or misincorporation frequency *f* for the control and modified templates were in the order dG > dC ≥ dT with all three polymerases. On the contrary, the relative efficiency *RF* of dCMP incorporation for HdU and HMdU lesion was almost doubly related to the control (Tables S1–S3). The most error-prone polymerase is polη (Figure 4A,D,G, Tables S1–S3).

To examine mutagenicity of HdU and HMdU pyrimidine derivatives more thoroughly, we express the relative dNMP mutagenicity as a ratio of the respective misincorporation frequency *f* = (*K*_cat_/*K*_m_)_incorrect_/(*K*_cat_/*K*_m_)_correct_ for the oligonucleotides with thymine derivatives and control oligonucleotide with thymine (Figure 4G–I, Tables S1–S3). In accordance with the literature data [2], dCMP was found to be the most preferable mismatch-nucleotide incorporated opposite the HdU lesion. The HMdU lesion was less mutagenic, which supports the view that HMdU incorporated in DNA may serve as an important epigenetic mark [11,13].

Collectively, TLS by DNA polymerases across the lesion derived from HdU was markedly less extensive and potentially more mutagenic than that across the lesion formed by HMdU. In other words, this report suggests that the replacement of methyl group in dT by hydroxyl group, capable of hydrogen bonding and electrostatic interactions with DNA and/or enzyme, resulting in the HdU lesion, appears to be a major factor responsible for the markedly lowered tolerance of this DNA lesion by DNA polymerases and its markedly higher mutagenicity. Moreover, HdU can form stable base-pairs with dA, dG, dC, and dT residues in double-helical DNA [3]. Moreover, dA and dC (in a different sequence context) were previously shown [2] to be preferentially incorporated opposite HdU lesion in DNA, thereby providing another basis for HdU enhanced mutagenicity.

## 3. Conclusions

The chemically simulated translesion DNA synthesis (TLS) across the lesions formed by HdU or HMdU was examined using microscale thermophoresis (MST). The thermodynamic changes associated with replication across HdU or HMdU show that the HdU paired with the mismatched deoxyribonucleoside triphosphates disturbs DNA duplexes considerably less than dT or HMdU. Moreover, we also demonstrate that TLS by DNA polymerases across the HdU lesion was markedly less extensive and potentially more mutagenic than that across the lesion formed by HMdU. These findings show that the significantly higher nucleotide misincorporation across the HdU lesion observed in this study for DNA polymerization by polη, KF^–^, and HIV-1 RT across these pyrimidine lesions plausibly correlated with the different stabilization effects determined by MST of the HdU and HMdU lesions in DNA duplexes.

The results of MST (Figure 2, Table 1, Table 2, Table 3 and Table 4) suggest that the lowest *K*_d_ values were found for the dissociation of duplexes formed between the 15mer DNA templates G**T**T(15) and G**X**T(15) (X = HdU or HMdU) and the matched or mismatched primers n or n+1 (where n = A nucleotide) compared to other combinations such as G:HdU, C:HdU or T:HdU. These results are consistent with those shown in Figure 4, which indicate that A is preferably incorporated opposite HdU lesion in DNA. The results of both MST and in vitro bypass assays also indicate that the HdU lesion in DNA may lead to a C to T mutation.

The *K*_d_ values found for incorporation of dAMP opposite to the HMdU lesion (n or n+1) in template DNA are much lower than those found for other dNMP incorporation (Table 1, Table 2, Table 3 and Table 4). Moreover, the results shown in Figure 3 suggest that DNA polymerases smoothly bypass HMdU lesions in the template DNA. The results of MST and in vitro bypass assays suggest that this lesion is less mutagenic and less inhibitory to DNA replication, consistent with the roles of HMdU in DNA as an epigenetic marker. Collectively, the results of this study also confirm the previously obtained results demonstrating HdU mutagenesis and the less-mutagenic nature of HMdU lesions with MST analysis.

Additionally, these thermodynamic data helps explain the role of the functional groups of DNA bases in the process of tolerance of DNA lesions, i.e., in the process related to the development of cellular damage and epigenetic signals. Additionally, the equilibrium thermodynamic data obtained by MST can possibly explain the influence of the thermodynamic alterations on the ability of DNA polymerases to bypass lesions induced by oxidative products of pyrimidines. Nevertheless, it is necessary to be aware of the fact that the kinetic effects may play an important role in the processing of the lesions formed by HdU or HMdU by DNA polymerases; the character of the polymerase may control the equilibrium between kinetic and thermodynamic effects.

The results also highlight the usefulness of MST in evaluating the impact of oxidative products of pyrimidines on the processing of these lesions by damaged-DNA processing-enzymes. Finally, the results of this work also expand the database correlating the thermodynamic characteristics of well-defined DNA damage and its mutagenic effects.

## 4. Materials and Methods

### 4.1. Chemicals

The synthetic oligodeoxyribonucleotides were purchased from Eurofins Genomics (Ebersberg, Germany). The synthetic oligodeoxyribonucleotides with HdU or HMdU modifications were obtained from TriLink biotechnologies (San Diego, CA, USA), repurified and/or extensively dialyzed against ultrapure water Milli-Q. Recombinant, full-length human DNA polymerase η (XPV protein) was purchased from EnzyMax, LLC (Lexington, KY), and reverse transcriptase from human immunodeficiency virus type 1 (HIV-1 RT) was from Calbiochem (San Diego, CA, USA). The exonuclease deficient Klenow fragment (KF^–^), T4 polynucleotide kinase, and dNTPs were purchased from New England Biolabs (Beverly, MA, USA). Acrylamide, bis(acrylamide), and urea were from Merck KgaA (Darmstadt, Germany). Radioactive products were from M.G.P. (Zlin, Czech Republic).

### 4.2. Purification of Oligonucleotides

The oligonucleotides containing single, site-specific HdU or HMdU instead of thymidine (5-methyluridine) were repurified by anion-exchange HPLC as described previously [49]. Concentration was determined by the measurements of the optical density.

### 4.3. Microscale Thermophoresis (MST) Determination of Thermodynamic Parameters of DNA Constructs with Nucleotide Misincorporation Opposite Pyrimidine Derivatives

MicroScale thermophoresis (MST) is a powerful technique to quantify biomolecular interactions. The biophysical background of MST was reported previously [42,50,51,52]. It is based on thermophoresis, the directed movement of molecules in a temperature gradient, which strongly depends on a variety of molecular properties such as size, charge, hydration shell or conformation. Thus, this technique is highly sensitive to virtually any change in molecular properties, allowing for precise quantification of molecular events independent of the size or nature of the investigated sample [42].

During an MST experiment, a temperature gradient is induced by an infrared laser. The directed movement of molecules through the temperature gradient was detected and quantified using covalently attached fluorophore (cyanine dye Cy5). Thermodynamic parameters were extracted from the temperature dependence of the dissociation constant, as shown by the following relations: Given a reversible, bimolecular reaction, L + M = LM, the dissociation constant *K*_d_ is defined as *K*_d_ = L × M/LM, which in turn directly depends on the Gibbs free energy change: Δ*G* = *RT*ln*K*_d_. Thus, by measuring *K*_d_s over a temperature range, Δ*G*, Δ*H*, and Δ*S* can be calculated. Since MST is a powerful method to precisely determine *K*_d_s with low sample consumption and on a short time scale, it is well suited for thermodynamic analyses of molecular interactions.

We measured the DNA-hybridization equilibrium binding constant of 15-nucleotide-long oligonucleotide templates and Cy5-labeled primers (Figure 2A) over a range of temperatures using a Monolith NT115^Pico^ device. DNA hybridization was readily detected by MST due to the different thermophoretic signals of single-stranded and double-stranded DNA. Instrument and settings: Monolith NT.115^Pico^ (NanoTemper Technologies GmbH, Germany) (https://nanotempertech.com/monolith/), MST power = medium (40%); LED power = 5–20% (10%). Cy5-labeled DNA primers were purchased from Eurofins Genomics. Measurements were carried out in the buffer (10 mM phosphate buffer, pH 7.0, 150 mM NaCl), supplemented with 0.05% Tween-20 and using standard capillaries. Photobleaching was suppressed using low LED power. Binding curves were obtained over a temperature range from 295 K to 308 K at 1–5 K increments regulated by the internal temperature control of the Monolith NT.115^Pico^ device; the accuracy of the temperature setting was ±0.5 °C. The concentration of the 5‘- Cy5 labeled DNA primers was kept constant at 2 nM, and its perfect match or one of three mismatches was diluted in a range from 10 µM down to 0.03 nM, respectively. For each temperature, MST measurements were started 180 s after reaching the desired temperature. The *K*_d_ values were calculated for each temperature by fitting the T-Jump signal or thermophoresis signal and plotted as ln(1/*K*_d_) vs. 1/*T* (K) in a van’t Hoff plot. Δ*H* was obtained from the slope m of the linear fit as m = −*H*^o^/*R*. Under the assumption that Δ*H* is constant in the relatively small linear range of the van’t Hoff plot Δ*S* was directly derived from the plot as y(0) = Δ*S*^o^/*R*. The universal gas constant *R* was calculated as 8.314 J K^−1^mol^−1^ [42].

### 4.4. Primer Extension Activity of DNA Polymerases

The 23mer templates (Figure 2A) containing a single lesion, major oxidative products formed by oxidation of pyrimidine residues HdU or HMdU were prepared as described above. 12mer (gap-primer for “running start” experiments) or 17mer (no gap-primer for “standing start” experiments) DNA primer (their sequence is shown in Figure 2A and Figure 3) were complementary to the 3’ termini of the 23mer templates. The DNA substrates (5 × 10^−8^ M) were formed by annealing templates and 5’-end-radiolabeled 12mer or 17mer primers at a molar ratio of 3:1. All experiments using polη or HIV-1 RT were performed at 310 K in 25 μL in a buffer containing 40 mM Tris-HCl (pH 8.0), 2 mM MgCl_2_, 10 mM dithiothreitol (DTT), 250 μg/mL bovine serum albumin (BSA), 60 mM KCl, 2.5% glycerol for polη (1.28 × 10^−8^ M, 1 ng/µL), ca. 1/4 enzyme/substrate and dNTPs, 100 μM each or 50 mM Tris-HCl (pH 8.0), 10 mM MgCl_2_, 50 mM KCl, 3 mM DTT, 0.1% Nonidet P-30, 2 U of the HIV1-RT enzyme, and deoxyribonucleotide 5‘-triphosphates (100 μM each). All experiments using KF^–^ were performed at 301 K in 25 μL in a buffer containing 50 mM NaCl, 10 mM Tris-HCl (pH 7.9), 10 mM MgCl_2_, 1 mM DTT, and 80 μg/mL BSA with KF^–^ (0.25 U) in the presence of all four deoxyribonucleotide 5‘-triphosphates. At appropriate time intervals, sample aliquots (5 uL) were withdrawn, and enzymatic reactions were terminated by the addition of LB den. (95% formamide, 0.3% bromphenol blue, and 0.3% xylene cyanol, 1× TBE) and/or EDTA so that its resulting concentration was 20 mM, and heating at 100 °C for 30 s. Products were resolved by denaturing 15% PAA/8 M urea gel and visualized by autoradiography.

The amount of TLS (translesion DNA synthesis) was defined as the amount of radioactivity corresponding to the products 20–23 nucleotides long on the 5’ side of the template strand and beyond divided by the total radioactivity in the lane. Other details were as published previously [53,54].

### 4.5. Analysis of Nucleotide Misincorporation Opposite Pyrimidine Derivatives

DNA polymerase fidelity on pyrimidine derivatives modified substrates was also tested. Experiments were performed under the same reaction conditions as primer extension kinetic studies of individual polymerases in the steady-state (60 min) in the presence of all four deoxyribonucleotide 5‘-triphosphates or selected dNTPs, complementary dATP, or non-complementary dCTP, dGTP, and dTTP (100 μM each). The amount of the “standing-start” synthesis was defined as the amount of radioactivity corresponding to the products of incorporation, 18–23 nucleotides long, on the 5’ side of the template strand and beyond division by the total radioactivity in the lane.

Steady-state kinetic analysis for individual dNTP incorporation opposite the thymine or its derivatives in 5′ G**X**T template sequences catalyzed by polη, KF^−^, or HIV-1 RT was done as described previously [29]. Briefly, appropriate polymerase was incubated with DNA substrates generated by annealing the 23-mer template to the 17mer 5′-^32^P labeled primer (Figure 4) with increasing concentration of the particular dNTP (0.5–500 µM) for 10 min under standard reaction conditions (vide supra). The percentage of primer extended was plotted as a function of the dNTP concentration, and the data were fitted by non-linear regression using GraphPad 7.04 software to the Michaelis–Menten equation describing hyperbola (Figures S3–S5). Apparent *K*_m_ and *V*_max_ steady-state parameters were obtained from the best fit and were used to calculate biochemical parameters (Tables S1–S3). The relative efficiency *RF* of the dNTP insertion opposite the HdU- or HMdU-modified templates was calculated as ((*K*_cat_/*K*_m_)_modif. template_/(*K*_cat_/*K*_m_)_unmodified template_). Relative dNTP mutagenicity (panels G, H, I) was calculated as a ratio of the respective misincorporation frequency *f* = (*K*_cat_*/K*_m_)_incorrect_/(*K*_cat_*/K*_m_)_correct_ for the oligonucleotides with thymine derivatives and control oligonucleotide with thymine.

### 4.6. Other Physical Methods

Absorption spectra were measured with a Beckmann DU-7400 spectrophotometer. For spectral analysis, purified oligonucleotides were dialyzed against deionized water Milli-Q. Purification of oligonucleotides with the aid of HPLC was carried out on a Waters HPLC system consisting of aWaters 262 Pump, Waters 2487 UV detector, and Waters 600S Controller with MonoQ HR 5/5 column. Gels were visualized using the Typhoon FLA 7000 bioimaging analyzer, and the radioactivity associated with bands was quantitated with AIDA image analyzer software (Raytest, Germany).

## Figures and Tables

**Table 1:** MST-derived thermodynamic parameters for the dissociation of duplexes formed between the 15mer DNA templates G**T**T(15) and G**X**T(15) (X = HdU or HMdU) and the matched or mismatched primers n or n+1, where n = A nucleotide.^1.^

Control Duplexes	Δ*H* (kJ mol^−1^)^2^	Δ*S* (kJ K^−1^ mol^−1^)^2^	Δ*G*_0_^310^ (kJ mol^−1^)^2^	*K*_d_^[310]3^	*K*_d_^[298]3^
G**T**T(15) n = A	367	1.090	29.4	11.38 µM	38.9 nM
G**T**T(15) n+1 n = A	377	1.104	34.3	1.66 µM	4.6 nM
Duplexes containing HdU lesions	Δ*H* (kJ mol^−1^)^2^	Δ*S* (kJ K^−1^ mol^−1^)^2^	Δ*G*_0_^310^ (kJ mol^−1^)^2^	*K*_d_^[310]3^	*K*_d_^[298]3^
G**X**T(15) n = A	354 (–13)	1.046 (–0.044)	29.9 (0.5)	9.12 µM	37.8 nM
G**X**T(15) n+1 n = A	362 (–15)	1.052 (–0.052)	35.3 (1.0)	1.15 µM	3.9 nM
Duplexes containing HMdU lesions	Δ*H* (kJ mol^−1^)^2^	Δ*S* (kJ K^−1^ mol^−1^)^2^	Δ*G*_0_^310^ (kJ mol^−1^)^2^	*K*_d_^[310]3^	*K*_d_^[298]3^
G**X**T(15) n = A	314 (–53)	0.917 (–0.173)	29.3 (–0.1)	11.44 µM	86.8 nM
G**X**T(15) n+1 n = A	330 (–47)	0.955 (–0.149)	33.2 (–1.1)	2.53 µM	14.9 nM

^1^ The nucleotide sequences of the templates and primers are shown in Figure 2A. ^2^ The Δ*H* and Δ*S* values are averages derived from two independent experiments. The uncertainties of the parameters are as follows: Δ*H* (±3%), Δ*S* (±3%), Δ*G*_310_ (±1%), *K*_d_[298] or *K*_d_[310] (±4%). “ΔΔ” parameters are in parentheses (these parameters are computed by subtracting the appropriate value measured for the control, the G**T**T duplex, from the value measured for the same duplex containing the single, site-specific HdU or HMdU lesion). Δ*G*_0_^310^ = Δ*H*−*T*Δ*S*; *T* = 310 K. ^3^ *K*_d_[310] or *K*_d_[298] denote the dissociation constants for strand dissociation at 310 or 298 K, respectively.

**Table 2:** MST-derived thermodynamic parameters of dissociation of duplexes formed between the 15mer DNA templates G**T**T(15) and G**X**T(15) (X = HdU or HMdU) and the matched or mismatched primers n or n+1, where n = G nucleotide.^1.^

Control Duplexes	Δ*H* (kJ mol^−1^)^2^	Δ*S* (kJ K^−1^ mol^−1^)^2^	Δ*G*_0_^310^ (kJ mol^−1^)^2^	*K*_d_^[310]3^	*K*_d_^[298]3^
G**T**T(15) n = G	290	0.851	25.6	48.22 µM	528 nM
G**T**T(15) n+1 n = G	308	0.905	26.9	29.54 µM	231 nM
Duplexes containing HdU lesions	Δ*H* (kJ mol^−1^)^2^	Δ*S* (kJ K^−1^ mol^−1^)^2^	Δ*G*_0_^310^ (kJ mol^−1^)^2^	*K*_d_^[310]3^	*K*_d_^[298]3^
G**X**T(15) n = G	271 (–19)	0.782 (–0.069)	28.4 (2.8)	16.64 µM	233 nM
G**X**T(15) n+1 n = G	322 (14)	0.950 (0.045)	27.7 (0.8)	21.74 µM	143 nM
Duplexes containing HMdU lesions	Δ*H* (kJ mol^−1^)^2^	Δ*S* (kJ K^−1^ mol^−1^)^2^	Δ*G*_0_^310^ (kJ mol^−1^)^2^	*K*_d_^[310]3^	*K*_d_^[298]3^
G**X**T(15) n = G	269 (–21)	0.780 (–0.071)	26.5 (0.9)	34.03 µM	522 nM
G**X**T(15) n+1 n = G	272 (–36)	0.790 (–0.115)	27.3 (0.4)	25.65 µM	378 nM

Footnotes 1–3 have the same meaning as those under Table 1.

**Table 3:** MST-derived thermodynamic parameters of dissociation of duplexes formed between the 15mer DNA templates G**T**T(15) and G**X**T(15) (X = HdU or HMdU) and the matched or mismatched primers n or n+1, where n = C nucleotide.^1.^

Control Duplexes	Δ*H* (kJ mol^−1^)^2^	Δ*S* (kJ K^−1^ mol^−1^)^2^	Δ*G*_0_^310^ (kJ mol^−1^)^2^	*K*_d_^[310]3^	*K*_d_^[298]3^
G**T**T(15) n = C	311	0.923	25.0	60.69 µM	484 nM
G**T**T(15) n+1 n = C	303	0.899	24.4	78.29 µM	687 nM
Duplexes containing HdU lesions	Δ*H* (kJ mol^−1^)^2^	Δ*S* (kJ K^−1^ mol^−1^)^2^	Δ*G*_0_^310^ (kJ mol^−1^)^2^	*K*_d_^[310]3^	*K*_d_^[298]3^
G**X**T(15) n = C	314 (3)	0.925 (0.002)	27.4 (2.4)	24.22 µM	173 nM
G**X**T(15) n+1 n = C	311 (8)	0.916 (0.017)	26.8 (2.4)	30.82 µM	244 nM
Duplexes containing HMdU lesions	Δ*H* (kJ mol^−1^)^2^	Δ*S* (kJ K^−1^ mol^−1^)^2^	Δ*G*_0_^310^ (kJ mol^−1^)^2^	*K*_d_^[310]3^	*K*_d_^[298]3^
G**X**T(15) n = C	299 (–12)	0.880 (–0.043)	26.5 (1.5)	34.45 µM	313 nM
G**X**T(15) n+1 n = C	304 (1)	0.899 (0)	25.3 (0.9)	55.54 µM	509 nM

Footnotes 1–3 have the same meaning as those under Table 1.

**Table 4:** MST-derived thermodynamic parameters of dissociation of duplexes formed between the 15mer DNA templates G**T**T(15) and G**X**T(15) (X = HdU or HMdU) and the matched or mismatched primers n or n+1, where n = T nucleotide.^1.^

Control Duplexes	Δ*H* (kJ mol^−1^)^2^	Δ*S* (kJ K^−1^ mol^−1^)^2^	Δ*G*_0_^310^ (kJ mol^−1^)^2^	*K*_d_^[310]3^	*K*_d_^[298]3^
G**T**T(15) n = T	290	0.858	24.3	81.14 µM	851 nM
G**T**T(15) n+1 n = T	293	0.865	24.2	82.57 µM	798 nM
Duplexes containing HdU lesions	Δ*H* (kJ mol^−1^)^2^	Δ*S* (kJ K^−1^ mol^−1^)^2^	Δ*G*_0_^310^ (kJ mol^−1^)^2^	*K*_d_^[310]3^	*K*_d_^[298]3^
G**X**T(15) n = T	276 (–14)	0.806 (–0.052)	26.3 (2.0)	36.83 µM	498 nM
G**X**T(15) n+1 n = T	303 (10)	0.897 (0.032)	24.8 (0.6)	65.54 µM	579 nM
Duplexes containing HMdU lesions	Δ*H* (kJ mol^−1^)^2^	Δ*S* (kJ K^−1^ mol^−1^)^2^	Δ*G*_0_^310^ (kJ mol^−1^)^2^	*K*_d_^[310]3^	*K*_d_^[298]3^
G**X**T(15) n = T	264 (–26)	0.770 (–0.088)	25.3 (1.0)	55.42 µM	873 nM
G**X**T(15) n+1 n = T	289 (–4)	0.855 (–0.010)	23.5 (–0.7)	109.91 µM	1.18 µM

Footnotes 1–3 have the same meaning as those under Table 1.

## Supplementary Materials

Supplementary material can be found at https://www.mdpi.com/1422-0067/20/20/5012/s1.

## Abbreviations

HdU	2’-deoxyribo-5-hydroxyuridin
HMdU	2’-deoxyribo-5-hydroxymethyluridin
TLS	translesion DNA synthesis
polη	DNA polymerase η
KF^–^	Klenow fragment of DNA polymerase I (the exonuclease deficient)
HIV-1 RT	reverse transcriptase from human immunodeficiency virus type 1
dT	thymidine
dNTP	2’-deoxyribonucleotide-5‘-triphosphate
PAA	polyacrylamide
MST	microscale thermophoresis
DSC	differential scanning calorimetry
DTT	dithiothreitol
BSA	bovine serum albumin
TET	ten eleven translocation
