# Supplementary material for: Translesion DNA Synthesis Across Lesions Induced by Oxidative Products of Pyrimidines: An Insight into the Mechanism by Microscale Thermophoresis
Source: Int J Mol Sci. 2019 Oct 10;20(20):5012. doi: 10.3390/ijms20205012 (PMC6829345; doi:10.3390/ijms20205012)
Supplement: Supplementary file 1 [file ijms-20-05012-s001.pdf]

Supplementary Material to:

Translesion DNA Synthesis Across Lesions Induced by Oxidative Products of Pyrimidines. An Insight into the Mechanism by Microscale Thermophoresis

Ondrej Hrabina [1,2], Viktor Brabec[1,2] and Olga Novakova [1,*]

[1] Czech Academy of Sciences, Institute of Biophysics, Kralovopolska 135, CZ-61265 Brno, Czech Republic; olga@ibp.cz, hrabina.ondra@gmail.com, brabec@ibp.cz

[2] Department of Biophysics, Faculty of Science, Palacky University, Slechtitelu 27, CZ7837177146 Olomouc, Czech Republic;

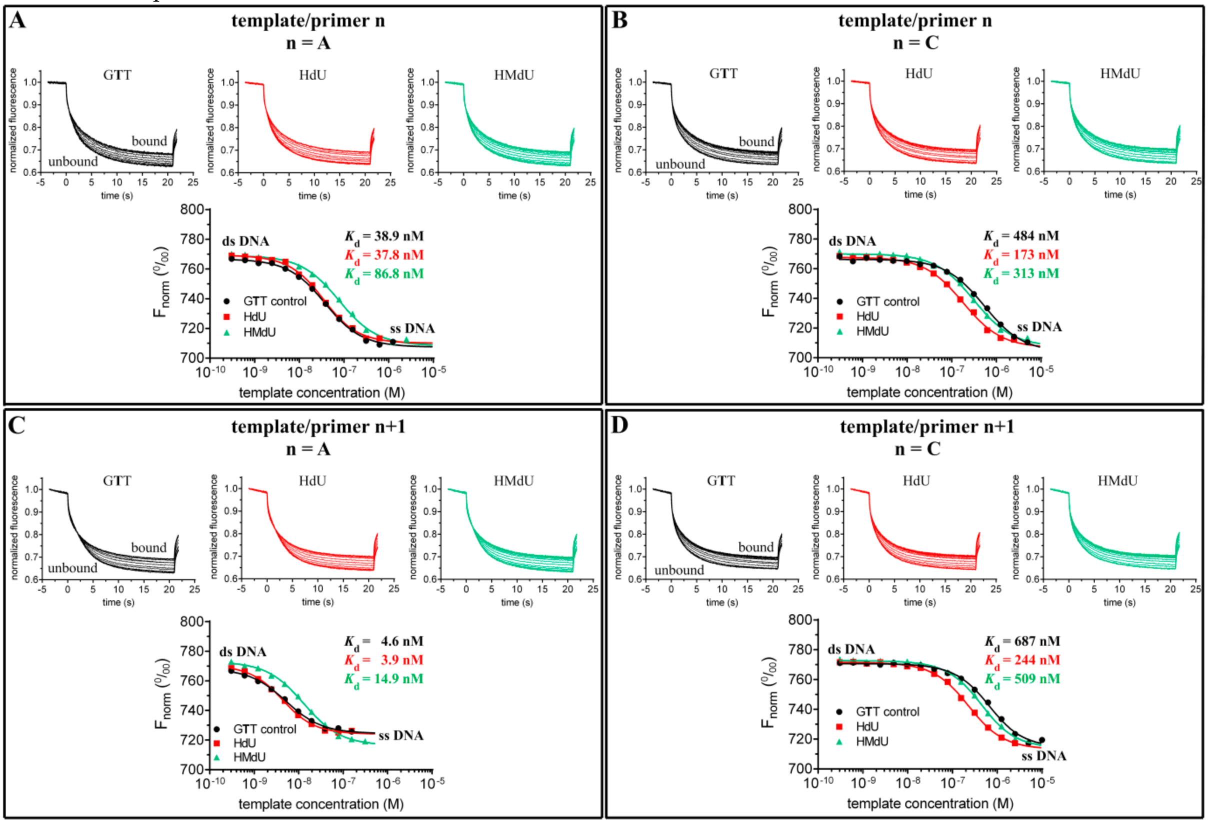

Figure S1. Exemplary MST time traces used to determine K_ds at 298 K for sets of the templates (containing thymine, HdU or HMdU lesion) hybridized with 2 nM primers n or n+1, where n is matched dA (Figures S1A,C) or mismatched dC (Figures S1B,D). MST timetraces corresponding to the titration of the template strand against Cy5-labeled primer are shown in top panels in Figures S1A, B, C, D: Black lines, undamaged templates; red and green lines, templates containing HdU or HMdU lesions, respectively, all normalized to a starting value of 1.0. Bottom panels in Figures S1A, B, C, D represent the change in MST signals that were fitted to yield K_d values. Single-stranded (ss) DNA yields a stronger MST response then double-stranded (ds) DNA.

Table S1. Kinetics of the incorporation of dNTP by human DNA polymerase η. The dNTP incorporation opposite the position 18 and extended (18+) in 23mer DNA template G**T**T(23) or G**X**T(23) (X = HdU or HMdU) which formed duplex with 17mer primer n – 1. n = complementary **A** or non-complementary G, C or T nucleotide in extended primer (18+).[1]

**Control duplex**	**V_{max} (% min^{-1})**	**K_{cat} (hour^{-1})**	**K_m (μM)**	**K_{cat}/K_m (μM^{-1}hour^{-1})**	**f^a**	**RF^b**
G**T**T(18+) n = **A**	9.96 ± 0.29	23.33 ± 0.68	0.43 ± 0.07	54.04	1	1
n = G	6.79 ± 0.77	15.92 ± 1.80	6.37 ± 2.30	2.50	0.04626	1
n = C	6.36 ± 1.06	14.89 ± 2.49	99.85 ± 28.13	0.15	0.00278	1
n = T	2.78 ± 0.27	6.52 ± 0.64	38.21 ± 9.04	0.17	0.00315	1

Duplex containing HdU lesion	V_{max} (% min^{-1})	K_{cat} (hour^{-1})	K_m (μM)	K_{cat}/K_m (μM^{-1}hour^{-1})	f^a	RF^b
G**X**T(18+) n = **A**	5.57 ± 0.18	13.06 ± 0.41	1.07 ± 0.13	12.25	1	0.2
n = G	5.38 ± 0.44	12.61 ± 1.02	4.46 ± 1.31	2.83	0.23103	1.1
n = C	3.31 ± 0.27	7.75 ± 0.64	29.71 ± 6.17	0.26	0.02122	1.7
n = T	2.33 ± 0.16	5.46 ± 0.36	31.66 ± 5.22	0.17	0.01388	1.0

Duplex containing HMdU lesion	V_{max} (% min^{-1})	K_{cat} (hour^{-1})	K_m (μM)	K_{cat}/K_m (μM^{-1}hour^{-1})	f^a	RF^b
G**X**T(18+) n = **A**	7.48 ± 0.48	17.52 ± 1.13	0.67 ± 0.07	26.11	1	0.5
n = G	6.03 ± 0.75	14.12 ± 1.75	8.04 ± 2.30	1.76	0.06741	0.7
n = C	4.20 ± 0.42	9.84 ± 0.10	34.49 ± 28.13	0.29	0.01111	1.9
n = T	1.90 ± 0.12	4.46 ± 0.29	28.2 ± 9.0	0.16	0.00613	0.9

[1] The nucleotide sequences of the templates and primers are shown in the Figures 3A, B, C or 4A.

[a] misincorporation frequency $f = (K_{cat}/K_m)_{incorrect}/(K_{cat}/K_m)_{correct}$

[b] relative efficiency *RF* compares the efficiency (K_{cat}/K_m) of the particular dNTP insertion opposite thymine derivatives HdU or HMdU in G**X**T(23) templates to the efficiency of the same dNTP insertion opposite thymine in G**T**T(23) template.

Table S2. Kinetics of the incorporation of dNTP by Klenow fragment of DNA polymerase I (the exonuclease deficient) (KF$^-$) opposite the position 18 and extended (18+). 23mer DNA template G**T**T(23) or G**X**T(23) (X = HdU or HMdU) formed duplex with 17mer primer n – 1. n = complementary **A** or non-complementary G, C or T nucleotide in extended primer (18+).[1]

**Control duplex**	**V_{max} (% min^{-1})**	**K_{cat} (hour^{-1})**	**K_m (μM)**	**K_{cat}/K_m (μM^{-1}hour^{-1})**	**f^a**	**RF^b**
G**T**T(18+) n = **A**	9.79 ± 0.17	19.97 ± 0.34	0.25 ± 0.07	81.29	1	1
n = G	10.64 ± 0.36	21.71 ± 0.72	3.67 ± 0.51	5.92	0.07280	1
n = C	10.98 ± 2.14	22.41 ± 4.36	71.93 ± 21.13	0.31	0.00384	1
n = T	8.33 ± 1.86	17.00 ± 3.79	442.5 ± 171.6	0.038	0.00047	1

Duplex containing HdU lesion	V_{max} (% min^{-1})	K_{cat} (hour^{-1})	K_m (μM)	K_{cat}/K_m (μM^{-1}hour^{-1})	f^a	RF^b
G**X**T(18+) n = **A**	5.55 ± 0.09	11.32 ± 0.19	0.35 ± 0.05	32.01	1	0.4
n = G	4.87 ± 0.12	9.94 ± 0.23	3.25 ± 0.31	3.06	0.09559	0.5
n = C	3.07 ± 0.33	6.26 ± 0.68	9.40 ± 2.87	0.67	0.02081	2.2
n = T	2.56 ± 0.42	5.22 ± 0.85	78.33 ± 30.32	0.067	0.00209	1.8

Duplex containing HMdU lesion	V_{max} (% min^{-1})	K_{cat} (hour^{-1})	K_m (μM)	K_{cat}/K_m (μM^{-1}hour^{-1})	f^a	RF^b
G**X**T(18+) n = **A**	8.79 ± 0.40	17.94 ± 0.82	0.31 ± 0.13	57.22	1	0.7
n = G	9.96 ± 0.42	20.33 ± 0.85	6.22 ± 0.84	3.27	0.05715	0.6
n = C	5.81 ± 0.75	11.85 ± 1.53	16.55 ± 5.04	0.72	0.01258	2.3
n = T	7.16 ± 1.07	14.62 ± 2.17	325 ± 87	0.045	0.00079	1.2

The footnotes [1, a, b] have the same meaning as those under the Table S1.

Table S3. Kinetics of the incorporation of dNTP by reverse transcriptase from human immunodeficiency virus type 1 (HIV-1 RT) opposite the position 18 and extended (18+). 23mer DNA template G**T**T(23) or G**X**T(23) (X = HdU or HMdU) formed duplex with 17mer primer n – 1. n = complementary **A** or non-complementary G, C or T nucleotide in extended primer (18+).[1]

**Control duplex**	V_{max} (% min^{-1})	K_{cat} (hour^{-1})	K_m (μM)	K_{cat}/K_m (μM^{-1}hour^{-1})	f[a]	RF[b]
G**T**T(18+) n = **A**	6.52 ± 0.39	7.24 ± 0.44	8.07 ± 1.57	0.90	1	1
n = G	0.89 ± 0.08	0.99 ± 0.088	44.00 ± 9.90	0.022	0.02444	1
n = C	0.33 ± 0.08	0.36 ± 0.087	167.40 ± 84.24	0.0022	0.00244	1
n = T	0.27 ± 0.09	0.30 ± 0.099	149.8 ± 131.1	0.0020	0.00222	1

Duplex containing HdU lesion	V_{max} (% min^{-1})	K_{cat} (hour^{-1})	K_m (μM)	K_{cat}/K_m (μM^{-1}hour^{-1})	f[a]	RF[b]
G**X**T(18+) n = **A**	2.06 ± 0.13	2.41 ± 0.15	7.30 ± 1.60	0.33	1	0.4
n = G	0.29 ± 0.03	0.33 ± 0.031	18.88 ± 5.46	0.017	0.05152	0.8
n = C	0.21 ± 0.04	0.24 ± 0.046	60.94 ± 34.93	0.0039	0.01182	1.8
n = T	0.30 ± 0.10	0.34 ± 0.115	100.5 ± 108.9	0.0034	0.01030	1.7

Duplex containing HMdU lesion	V_{max} (% min^{-1})	K_{cat} (hour^{-1})	K_m (μM)	K_{cat}/K_m (μM^{-1}hour^{-1})	f[a]	RF[b]
G**X**T(18+) n = **A**	6.13 ± 0.45	6.82 ± 0.50	8.31 ± 1.94	0.82	1	0.9
n = G	1.29 ± 0.24	1.44 ± 0.27	78.96 ± 30.94	0.018	0.02195	0.8
n = C	0.44 ± 0.12	0.48 ± 0.135	162.30 ± 86.89	0.0030	0.00366	1.4
n = T	0.57 ± 0.30	0.64 ± 0.340	254 ± 264	0.0025	0.00305	1.3

The footnotes [1, a, b] have the same meaning as those under the Table S1.

* hour^{-1} was used because of too low HIV-1 RT processivity.

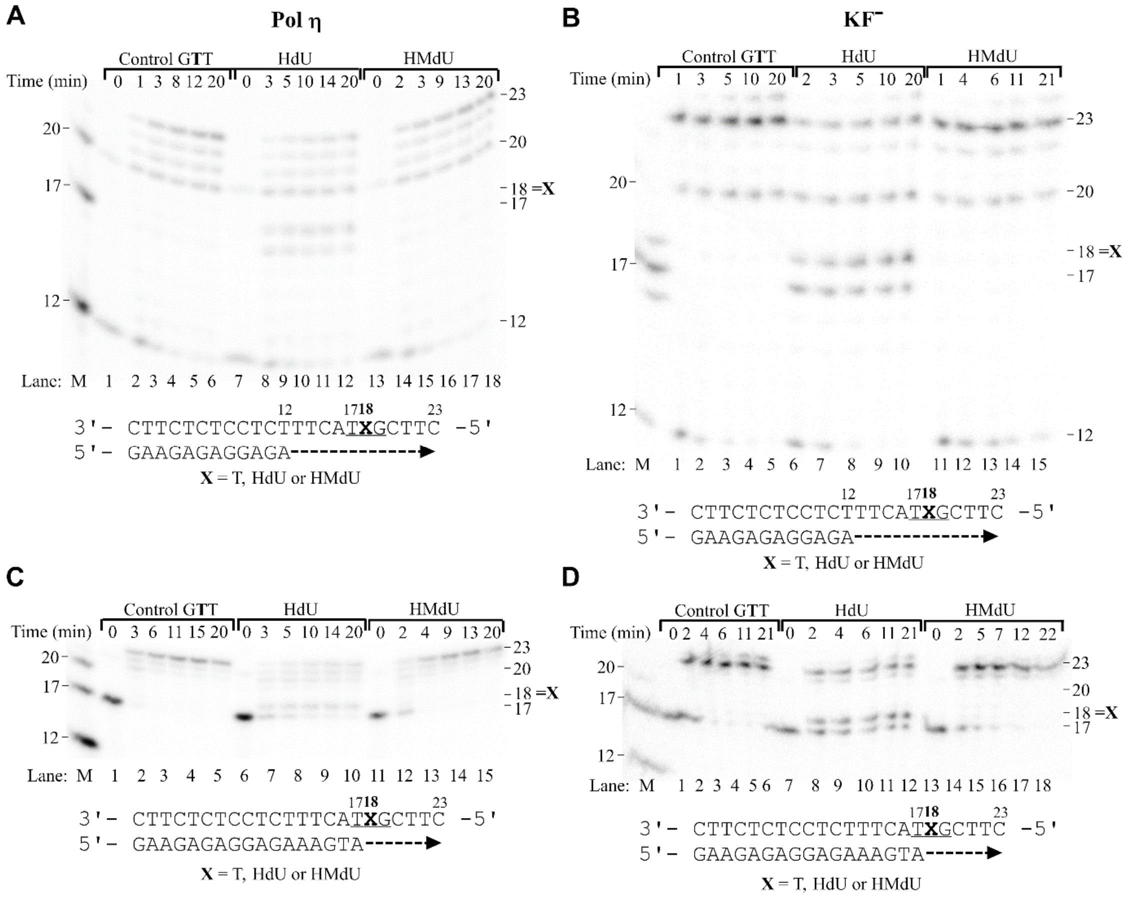

Figure S2. Translesion DNA synthesis by human DNA polymerase η (polη), and Klenow fragment of DNA polymerase I (the exonuclease deficient) (KF^-) on templates containing a site-specific thymine mutation. Primer extension activity of polη (A, C) and KF^- (B, D). (A, B, C, D) Representative images of the products of DNA polymerases reactions resolved on 15% PAA gels. 12mer (gap-primer for "running start" experiments) or 17mer (no gap-primer for "standing start" experiments). The experiments were conducted using the 12-mer/23-mer (panels A, B) or 17-mer/23-mer primer/template duplexes (panels C, D) for the various times (time points of 1 - 22 min are shown above the gels) using undamaged template, the template containing HdU or HMdU instead of thymine at the 5′-GXT sequence. The pause sites and position of thymine modification (the product lengths) are shown on the right side of the gels. Lane M: DNA markers. The nucleotide sequences of the templates and the primers are shown at the bottom of the panels A, B, C, D.

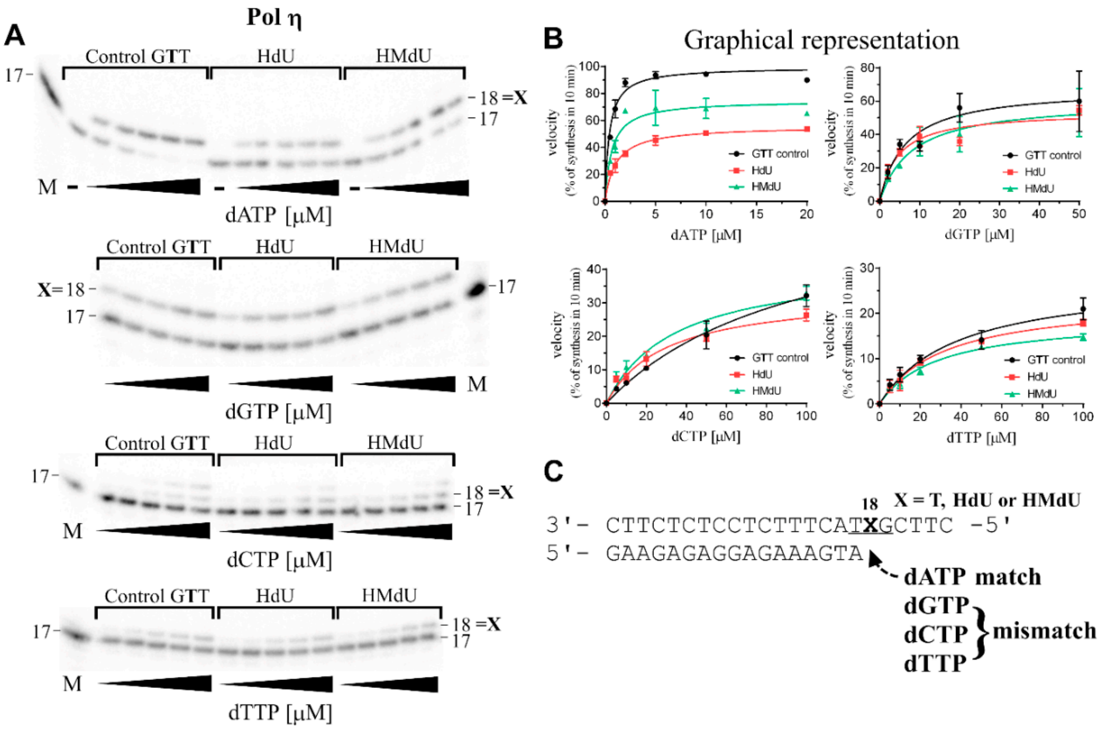

Figure S3. Representative gels for steady-state kinetic analysis of single nucleotide incorporation across **X** at the 5′-GXT sequences by polη (A). Reactions were done for 10 min and varying concentrations of the indicated dNTP (0.5 – 100 μM). Lane M: DNA markers. Steady-state kinetic analysis - graphical representation of individual dNTP insertions (B). Data are means (±SD) from at least two different experiments. Fitting was to a hyperbolic equation in GraphPad Prism v. 7.04, and V_{max}, K_{cat} and K_m values are presented in Tables S1-S3. The nucleotide sequences of the template and the primer (C).

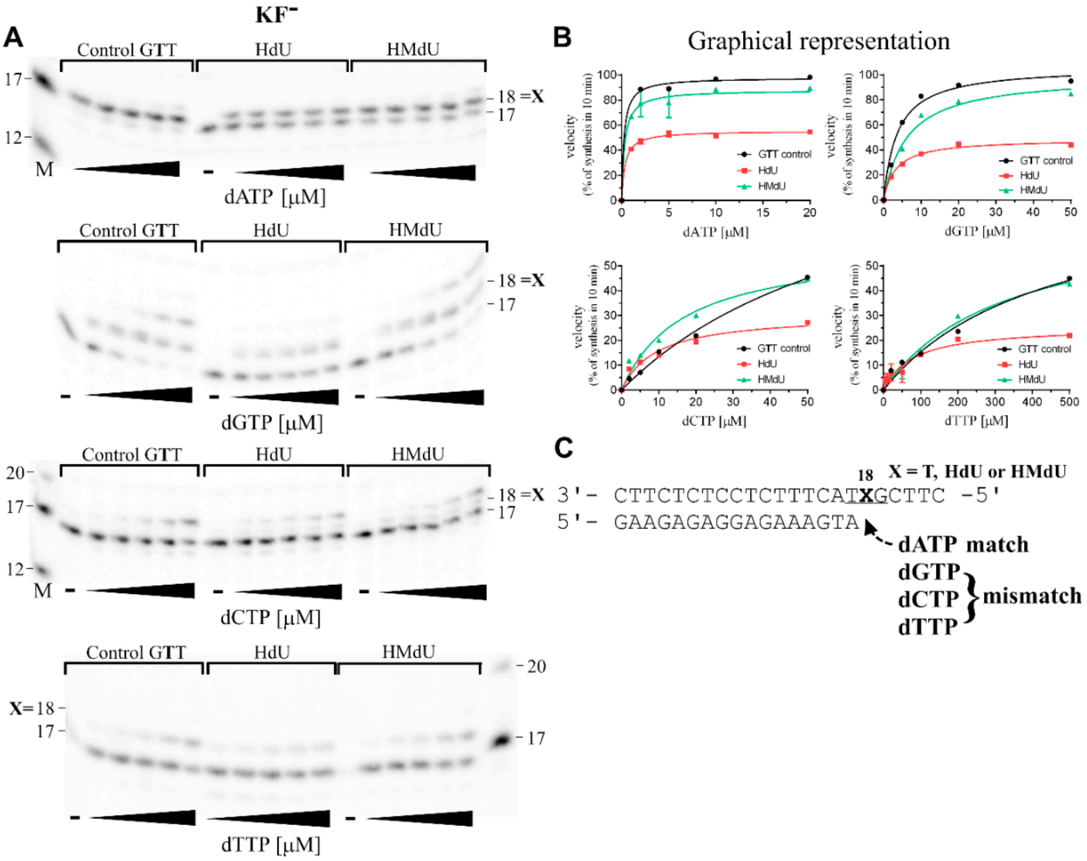

Figure S4. Representative gels for steady-state kinetic analysis of single nucleotide incorporation across **X** at the 5′-GXT sequences by KF^{-} (A). Reactions were done for 10 min and varying concentrations of the indicated dNTP (1 - 500 μM). Lane M: DNA markers. Steady-state kinetic analysis - graphical representation of individual dNTP insertions (B). Data are means (±SD) from at least two different experiments. Fitting was to a hyperbolic equation in GraphPad Prism v. 7.04, and V_{max}, K_{cat} and K_m values are presented in Tables S1-S3. The nucleotide sequences of the template and the primer (C).

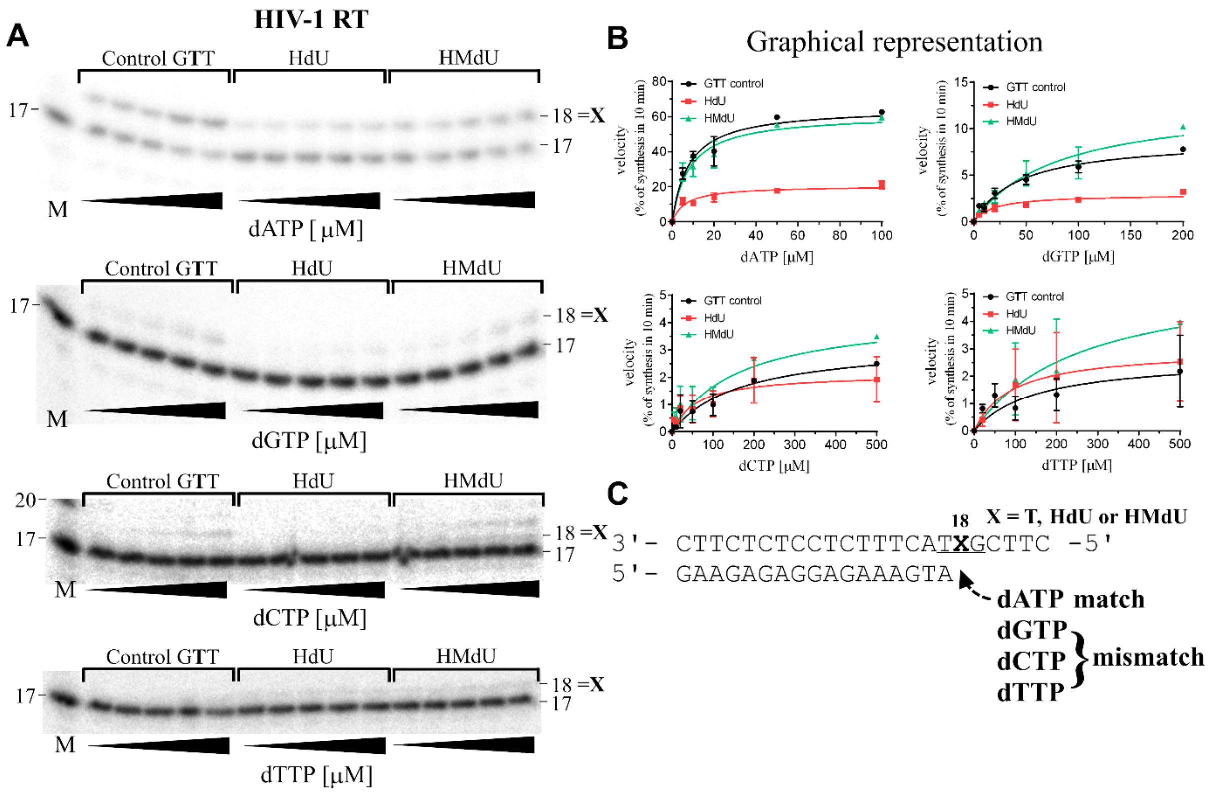

Figure S5. Representative gels for steady-state kinetic analysis of single nucleotide incorporation across **X** at the 5′-GXT sequences by HIV-1 RT (A). Reactions were done for 10 min and varying concentrations of the indicated dNTP (5 - 500 μM). Lane M: DNA markers. Steady-state kinetic analysis - graphical representation of individual dNTP insertions (B). Data are means (±SD) from at least two different experiments. Fitting was to a hyperbolic equation in GraphPad Prism v. 7.04, and V_{max}, K_{cat} and K_m values are presented in Tables S1-S3. The nucleotide sequences of the template and the primer (C).
